# Supplementary material for: Assessing swallowing disorders in adults on high-flow nasal cannula in critical and non-critical care settings. A scoping review protocol
Source: PLoS One. 2023 Oct 9;18(10):e0291803. doi: 10.1371/journal.pone.0291803 (PMC10561841; doi:10.1371/journal.pone.0291803)
Supplement: Attachment — Submitted filename: Response to Reviewers.docx [file pone.0291803.s002.docx]

**Response to Reviewers**

| **Comments** | **Responses** |
| --- | --- |
| **Journal Requirements** | |
| 1. Please ensure that your manuscript meets PLOS ONE's style requirements, including those for file naming… | The manuscript was edited according to PLOS ONE style requirements. |
| At this time, please address the following queries:  a) Please clarify the sources of funding (financial or material support) for your study. List the grants or organizations that supported your study, including funding received from your institution.  b) State what role the funders took in the study. If the funders had no role in your study, please state: “The funders had no role in study design, data collection and analysis, decision to publish, or preparation of the manuscript.”  c) If any authors received a salary from any of your funders, please state which authors and which funders.  d) If you did not receive any funding for this study, please state: “The authors received no specific funding for this work.”  Please include your amended statements within your cover letter; we will change the online submission form on your behalf. | In the “Rebuttal Letter” we requested the change of the funding statement to “The authors received no specific funding for this work.” |
| 3. Your ethics statement should only appear in the Methods section of your manuscript. If your ethics statement is written in any section besides the Methods, please move it to the Methods section and delete it from any other section. Please ensure that your ethics statement is included in your manuscript, as the ethics statement entered into the online submission form will not be published alongside your manuscript. | The ethics statement was moved to the Methods section and removed from other parts of the manuscript. |
| 4. Please include captions for your Supporting Information files at the end of your manuscript, and update any in-text citations to match accordingly. Please see our Supporting Information guidelines for more information: http://journals.plos.org/plosone/s/supporting-information. | Our Supporting Information subheading was included at the end of the manuscript and cited in the Methods section. |
| **Reviewer #1** | |
| Please descibe in full the methodology. Will any reference manager be used? What is the time frame? How is the distinction between critical and non-critical setting done? Are there two or three researchers engaged in the analysis of publications? | Thank you for your questions. Several methodological clarifications have been added to the manuscript:  ** “Once the studies had been searched, duplicates will be removed using the Mendeley® (Mendeley Desktop Version 1.19.8 - Elsevier Inc.) reference manager and the Rayyan® app”.*  ** “Data extraction will be carried out by 4 researchers in pairs of one experienced and one novice reviewer”.*  ** “Findings will be reported for all studies and separated by those conducted in critical and non-critical settings. The critical setting will comprise the intensive critical units, and the non-critical setting will comprise all other units and the out-of-hospital”.* |
| Figure 1 is not mentioned int the text, it also does not add any value to the publication. | Thank you for your comment. We have replaced the figure with a table to detail the data that will be extracted from the included studies. |
| The protocol requires english editing. | The protocol was reviewed and edited by a native English speaker. |
| Line 32 and line 33 - two consecutive sentences start with "However". Please replace it with a different vocabulary. | Thank you for your comment. *“However”* has been changed to *“Nevertheless”*. |
| Line 40 - the authors declare the research in any language will be considered. How are the authors planning to perform translation to understant the full context of the publication? | Thank you for your comment. In the Methods section, the following idea was added:  *“For the translation of studies published in languages other than English and Spanish, researchers who are native speakers of the language of publication will be contacted to resolve doubts regarding the general context of the study.”* |
| Line 44, line 166 - the term "hurt" seems to be ambiguous, please consider different term. | Thank you for your comment. |
| Line 45-48 - this sentence is too long and unclear, please consider rephrasing. | The term *"hurt"* was changed to *"impair"* in both sections of the manuscript. |
| Line 56 - please consider adding a subject, e.g "especially in patients with hypoxemic respiratory failure" instead of effectivenes in [...], especially in hypoxemic respiratory failure | Thanks for the suggestion. The sentence was replaced by *“A number of studies have reported the effectiveness of HFNC”*. |
| Line 59 - please consider using a different term instead of "known", "set" or "planned" seems to be more adequate verb here | Thanks for the suggestion. *“K*nown*”* has been changed to *“planned”*. |
| Line 63 0 please consider adding "its" or "HFNC" before the word "use" | Thanks for the suggestion. Added the term *“its”*. |
| Line 70 - none of the cited studies [19-21] concerns newborns. In study 19 & 20 pediatric population is being studied, however very few newborns are included. Study 21 reffers mainly to adults. | Thank you for your comment. Reference 21 was deleted, and the sentence was left as follows:  *“Most of the research related to the research question posed in this protocol has focused on pediatrics, probably because the evidence supports the use of HFNC in this publication.”* |
| Line 76 - please refrain from repeating an adjective "different" in one sentence. | Thank you for your comment. *“different”* has been changed to *“*diverse*”*. |
| Line 77-78 - the sentence "The flow used in HFNC needed to trigger them in adults with and without diseases in incomprehensible | The phrase was deleted. |
| Line 98 - 99 - repeated phrase "through the Ovid platform" | According to the PRISMA-S and PRESS declaration, the search platform is required for each database. In this case, we report that we will use OVID to search MEDLINE and Embase, as well as other platforms for the remaining databases. |
| Line 116-118 phrase "adults [...] who may be healthy or have a disease requiring acute or chronic use of HFNC" is unclear. Are patents requiring HFNC without any underlying diseases or with acute or chronic resp. diseases included? Please clarify | Thank you for your comment. Let us explain the next sentence better:  *“These may be healthy people who have voluntarily participated in studies to evaluate the use of HFNC or have a disease requiring acute or chronic use of HFNC.”* |
| Line 166-167 - "altering the preathing and swallowing pattern" - is altering the synchronization of breathing and swallowing ment? | Thanks for the suggestion. *“Altering the breathing and swallowing pattern”* was changed by *“synchronization of breathing and swallowing.”* |
| Line 168-170 - the sentence "This could be explained [...]." is unclear, the term "vitellus" stands for an egg yolk, what was ment? | We change the term *"vitellus"* to *“vellum”*. |
| **Reviewer #1** | |
| Some confoundings are commented: flow, oxygen fraction, device, location of patients (critical vs non-critical), age. My main concern is about management of these confoundings to present the results. Could authors detail? | The purpose of a scoping review is to describe what the literature says or what research has been done on a specific topic. Our aim is not to provide recommendations for clinical practice, which is rather the aim of systematic reviews and clinical practice guidelines, but to identify what has been studied regarding the possible association between the use of HFNC and swallowing disorders. Therefore, our study will serve to identify gaps in knowledge and provide recommendations for future research.  All possible variables that could confound the relationship between swallowing disorder and HFNC use will be discussed for future primary studies to consider. |
| Authors state that aspiration pneumonia has been associated with HFNC, but a cite is lacking. | A reference was added to support the possibility that the use of HFNC may generate aspiration. |
